# The Evidence of Cooperative Binding of a Ligand to G4 DNA

**DOI:** 10.1155/2017/6780521

**Published:** 2017-10-18

**Authors:** A. G. Kudrev

**Affiliations:** Saint Petersburg State University, 7/9 Universitetskaya Nab., St. Petersburg 199034, Russia

## Abstract

Intrinsic constants of the ligand binding with G4 DNA (guanine-rich DNA sequence) using quantitative standards can be convenient providing the assessment for elucidating the possibility of such structures participation in biochemical processes. In the present communication, the hard + soft modelling approach to calculate intrinsic constants of a ligand binding with short DNA molecule, particularly such as G4 DNA, has been proposed. The suggested approach has focused upon the quantitative evaluating of a mutual influence between sites and between bound ligands. The cross-validation between a new hard + soft modelling and conventional stepwise complex formation algorithm has been conducted. A number of simulated examples will illustrate the methodology. The experimental mole-ratio titration of TMPyP4 by G4 DNA [(CG_3_)_2_CGC(AG_3_)_2_G] has been reexamined. The [(CG_3_)_2_CGC(AG_3_)_2_G] that folds from a G-rich sequence found in the promoter region of c-kit oncogene can be considered as a molecule with two equivalent mutually influence binding sites.

## 1. Introduction

In the past decade, a great research effort around four-stranded guanine-rich DNA sequences known as G4-quadruplexes (G4) [1] and small molecule ligand interactions was motivated as both innovative basis for the design of novel inhibitors of telomerase activity [2] and selective fluorescent probes [3]. Recent bioinformatics studies have shown that in the human genome there are approximately 350,000 sequences that can potentially form G4 structures [4]. The current interest in G4 interactions is based on the concept that small molecules binding would stabilize these structures' formation, which causes an inhibition of telomerase activity, which plays a role in the immortal growth of cancer cells. Such small molecules-ligands are promising as novel anticancer drug candidates. Information in huge number of publications would be convenient to systematize using the stability of the complexes formed by the components of the systems in question or by the ability of ligand to bind G4 molecules present in the solution. However, in the present, such systematization is difficult to perform. The lack of reliable, quantitative characteristics for G4-quadruplexes binding properties complicates the development of bioinformatics tools, particularly for the analysis of involvement G4 in the inhibition of telomerase activities* in vivo* and for the discovery of new anticancer agents. To address this issue, robust binding models are needed to investigate G4-ligand interactions.

The TMPyP4 (5,10,15,20-tetrakis-(1-methyl-4-pyridyl)-21H,23H-porphyrin) is one ligand shown to be capable of binding to the G4-structures. The interaction between TMPyP4 and G4 has been extensively studied since it can inhibit the activity of telomerase upon binding to human telomeric sequences [5]. However, its binding mode is still controversial [6]. This cationic porphyrin contains a large *π*-planar structure and cationic functional groups providing strong *π*-*π* stacking and electrostatic interactions with G-quartets and phosphate groups on the G4 DNA. The variations in the UV-Vis absorption and fluorescence spectra of the binding TMPyP4 ligand are normally the result of a change in the molecular conformation of the ligand in addition to the change in the environmental polarity and strong transition moment dipole coupling between the base pairs of DNA and the ligand. The cationic porphyrin G4 interactions have been studied extensively using a wide variety of spectroscopic techniques and isothermal titration calorimetry (several examples reported previously are summarized in Table  S1; see Table S1 in the Supplementary Material available online at https://doi.org/10.1155/2017/6780521). The UV-visible optical spectroscopy (UV-VIS), fluorescence resonance energy transfer (FRET), and circular dichroism (CD) via the spectral changes provide crucial information about ligand G4 DNA interactions, such as stoichiometry, stabilization strength, and especially the selectivity for G4 in comparison to ds DNA [7]. However, the detail of the binding mode remains to be completely elucidated. Several models of the binding mode have been proposed to date, especially for intramolecular quadruplexes from human telomeric sequence [8].

The nature of the G4 DNA binding site is unmistakably crucial for understanding the binding stoichiometry which is one of the most important hard modelling parameters. The TMPyP4 planar molecule favours *π*-stacking with G-quartets, including end-stacking and intercalation, but also groove binding, and direct coordination to bases or the phosphate backbone is possible [7]. The cationic properties of TMPyP4 allow electrostatic interactions with the negative backbone of G4 DNA. In addition, the coexisting binding modes have been recently revealed [9]. As the porphyrin concentration increased, TMPyP4 started to stack along the DNA. The central flat part and peripheral N-methylpyridyl moiety of TMPyP4 have a common structure for self-stacking and intercalation; hence the patterns of shifts in UV-Vis and CD spectra were almost identical in the mentioned binding modes [10]. For either intercalation or end-stacking the stoichiometric ratio of ligand/G4 was found to be 2 : 1 in most cases [11]. However for an intramolecular antiparallel-parallel hybrid structure [d(AG3(T2AG3)3)], the weak anticooperativity of TMPyP4 accommodation onto two from three nonidentical sites was detected [12]. A complete picture is thus obtained, revealing the coexisting binding modes of intercalation and surface binding [12]. Subsequently, we can more precisely predict binding mode if in addition to the patterns of shifts in UV-Vis and CD spectra the stoichiometry and intrinsic binding constants are determined from experimental data.

The evaluation of intrinsic binding constants which belong to the G4 DNA-ligand-binding site seems to be the first important step toward understanding the ligand exertion of influence on G4 DNA. These interactions are difficult to monitor because of complex structures and topologies of G4-quadruplexes, different sites and potential modes of binding, and the presence of other secondary nucleic acid structures. The problem is related to the high and very dynamic topological plasticity of quadruplexes, and it is very difficult to study the binding of high-affinity ligands without evoking the great topological consequences of the ligands binding that in turn strongly influence the second binding event. Quantitative description of interaction G4 DNA with TMPyP4 has been performed using either the direct fit method [13] or the independent binding sites model [14] or the conventional stepwise complex formation model [15]. All these methods do not take into account the mutual influence (MI) in the course of successive ligand binding. Reasonably in addition to the different classes of binding sites, the cooperativity of interactions should be taken into account [16].

The thermodynamic effect of cooperativity may be due to changes in the macromolecule's conformation or redistribution of the electron density at the site due to the influence through oligomers bonds or by the MI between bound ligands via space [17]. A variety of methods have been developed for computational explanation of cooperative interactions of a ligand with set of binding sites [18]. If these sites are equivalent and independent, relevant experimental data may be analysed toward the thermodynamic binding constant and the number of binding sites in terms of a linear Scatchard plot [19]. Since this presumes a most simple binding model, such an approach is susceptible to substantial misinterpretations in practice [20].

The McGhee and von Hippel model which takes into account the MI between nearest neighbouring bound ligands has been the most widely used [21] in combination with the Scatchard equation for description of small molecules with homogeneous polymers. Sometimes those equations are incorrectly used to describe interactions with G4 and other DNA nonhomogeneous oligomers even though the authors of the model have concluded that the binding of ligands known to have heterogeneous binding sites on the DNA cannot be adequately treated based on the conditional probability approach [21]. Although Scatchard analysis is very much attractive owing to pictorial rendition, it has serious implications of unreliability regarding the shape of a Scatchard plot and it is evident that this method is not appropriate for quantitative evaluation of the binding of TMPyP4 with G4. The Job plots analysis of the ligand binding with the G-quadruplex sites accounting for their MI has been described in [12].

To compare the interpretation of spectrometrically measured data using the canonical stepwise model with that of mutually influencing binding sites, we considered the combination of hard model-based fitting and soft model-free Singular Value Decomposition (SVD) [22]. The Rank Annihilation Factor Analysis (RAFA) has also aided in unambiguous revealing of the cooperative interactions. This solution should improve our understanding of the mechanism of the G4 folding and binding at a molecular level, which in turn will help in highly efficient and selective telomerase inhibitors discovery.

## 2. Computational Details

### 2.1. Description of the Model

To simulate G4-ligand interactions the model based upon a multiple set of binding sites has been proposed [12]. When binding with G4 DNA is encountered, then each monomeric ligand is assumed to be bound to one of the sites on the DNA oligomer. The cooperativity is the enhancing of binding affinity in comparison with the sole binding, but anticooperativity is the reduction of binding affinity. The secondary interaction is an important source of ligand binding cooperativity. In DNA-ligand complexes, the arrangement of the ligands decides the total stability of the complex and elucidates that secondary interactions between neighbouring ligands can contribute additional stabilization [23]. To find a sign of cooperative interactions just the influencing of ligand on another ligand bound in the close vicinity has been taken into account. Consider intrinsic constants which govern the binding of ligand to a set of sites. In general case, the binding of *N* ligands among *N* vacancies gives 2^*N*^ possible variants of coordination. The one-dimension matrix of site-specific equilibrium constants **K**_1–**N**_ (**K**_1–**N**_ are constants for a ligand binding to each isolated site 1 ⋯ *N*) and matrix **Ω** of the MI corrections (cooperativity parameters $\left[\begin{array}{cccc} \omega_{1} & \omega_{2} & \cdots & \omega_{N} \end{array}\right]$) are constructed in accordance with hypothesis about structure of adducts formed in DNA oligomer saturation by a ligand. The values of **K**_1–**N**_ and **ω**_**i**_ could be found simultaneously as was described previously [24].

We will start here with looking at the simplest of situations for ligand binding, namely, the interaction with bidentate oligomer. The matrix of configurations **M** has been introduced in order to describe the detailed set of configurations adopted by the macromolecule in microstates. Each line of the matrix **M** displays a microstates configuration through a sequence of zeroes in those positions where the sites of multidentate macromolecule are not occupied by a ligand, and with a sequence of ones in those positions where the sites are bound to the ligand. In the course of the macromolecule sites saturation, previously bound ligands can affect the binding of the following ligands. For the oligomer containing two nonidentical binding sites, the matrix **M**^**∗**^ can be written as

(1) $$\begin{array}{c} M^{*}=\left[\begin{array}{cc} 0 & 0\\ K_{1} & 0\\ 0 & K_{2}\\ K_{1}\omega & K_{2}\omega \end{array}\right]. \end{array}$$

The product of the nonzero elements in **M**^**∗**^ provides the column of stability constants of microscopic species

(2) $$\begin{array}{c} B=\overset{N}{\underset{j=1}{\prod }}\left(\;M^{*}-M+E\;\right); \end{array}$$

here the matrix **E** is all ones and the same size as **M**. The estimates of the true nonlinear variables **K**_1-2_ and **ω** allow calculating the matrix **C**_**DNAL**_ of DNA oligomer species of concentration for the *i*th total concentration of ligand and DNA, correspondingly *C*_*L*_ and *C*_*D*_.

(3) CDNAL=DNA1DNAL′1DNAL′′1DNAL21⋯⋯⋯⋯DNANpDNAL′NpDNAL′′NpDNAL2Np.

Here [**DNA**] = **C**_**D**_(1 + [*L*]^**s**^**B**)^−1^ is an equilibrium concentration of free DNA oligomer and [DNA*L*_*s*_] = [DNA][*L*]^*S*^**B**^*T*^ are G4 DNA species equilibrium concentrations; [*L*] is an equilibrium concentration of free ligand; *S* = 1,2 is a stoichiometric coefficient; Np is the number of experimental points. In our case the sum of microforms equilibrium concentrations [DNA*L*′] and [DNA*L*′′] gives the concentration of oligomer species corresponding to single bound ligand. This species in conventional stepwise complex formation model is regarded as the 1 : 1 complex. Once we know **C**_**DNAL**_, we can calculate concentrations of free sites and these bound with a ligand.

(4) $$\begin{array}{c} C_{MonL}=\left[\begin{array}{cc} C_{DNAL}\left[E-M\right] & C_{DNAL}M \end{array}\right]. \end{array}$$

### 2.2. The Validation of the Proposed Model

According to Lambert–Beer's law, a spectrometrically measured data matrix **A** is the product of a concentration matrix **C** and a matrix **S** of molar absorptivities of species that contribute to the absorption of light at any wavelength (species pure spectra). The corresponding general matrix equation is given as

(5) $$\begin{array}{c} A=CS+\tau ; \end{array}$$

here **τ** is a matrix of residuals. The number of rows in matrix **A** is equal to the number of studied solutions with different components concentrations. The number of columns in matrix **A** is equal to the number of wavelengths. To simulate the data matrix **A** a concentration matrix **C** = **C**_**MonL**_ has been computed in accordance with equilibrium model of two-step ligand binding. The total light absorbance is due to the absorption of the occupied Mon_1_*L*, Mon_2_*L* and unoccupied sites Mon_1_, Mon_2_ of oligomer molecules and absorption of free and coordinated ligand *L*. The data set was designed to simulate an interaction ligand with oligomer (TMPyP4 with G-quadruplex DNA), which has two binding sites. Since the rank *R* of data matrix **A** is equal to the rank of **C** or **S**, whichever is smaller, and since the rank of either **C** or **S** can be no larger than the total number of spectral species, the rank of **A** can be no larger than the total number of spectral species, provided the number of wavelengths and the number of studied solutions are both greater than the number of spectral species. For simplicity in the current simulations residuals **τ** were put to zero. Thus the rank *R* is the number of singular values of **AA**^*T*^ that are bigger than machine precision (10^−10^–10^−13^).

### 2.3. The Least Square (LS) Fitting Procedure

The fitting procedure should deconvolve the data matrix **A** into matrices of concentrations **C** and molar absorptivities of a matrix **S**. Multiplication of both sides of (5) on a pseudoinverse [22] matrix **C**^+^ = (**C**^**T**^**C**)^−1^**C**^**T**^, computed with an initial estimate of the required parameters, gives a current estimation **S**^*∗*^ of the true matrix of molar parameters **S**. The best set of linear parameters **S**^*∗*^ for system in question can be calculated explicitly in a linear least-squares calculation by

(6) $$\begin{array}{c} S^{*}=C^{+}A. \end{array}$$

In order to evaluate linear as well as nonlinear parameters for a chemical model such as that described above, a decomposition of the experimental data matrix **A** is executed under a nonnegativity constraint. The product of **S**^*∗*^ and **C**^+^ gives **A**_**calc**_ at the current values for nonlinear parameters. Consequently, the iteration formula for refinement of the required nonlinear parameters can be written as

(7) $$\begin{array}{c} A_{calc}={\left[\;C^{+}A\;\right]}_{>0}C^{*}+\tau , \end{array}$$

where [⋯]_>0_ is the operator that substituted negative values to zero values (nonnegativity constraint only for spectral profiles was applied); **τ** is the residual matrix with unexplained data variance (here *τ* is machine precision). Relevant experimental data should be analysed toward the intrinsic association binding constants **K**_1-2_ and intrinsic parameter of cooperativity **ω** to obtain a best fit to the data. When optimal nonlinear parameters were found, the pure species spectra have been calculated in accordance with (6).

The Levenberg–Marquardt nonlinear LS algorithm is used for refinement of parameters [25]. The iterative procedure described above is used to find the minimum of the sum of squares of deviations of all absorbance data matrix elements which were calculated using theoretical model, from the corresponding matrix considered on the current iterative step, calculated in turn by using (7). Refinement was stopped when the relative difference between consecutive iterations fell below a threshold value. The Hamilton factor was used in the present work to compare matrices by applying the following equation:

(8) PEFexp,Fcalc=TraceFexp−FcalcFexp−FcalcTTraceFexpFexpT×100%,

where Trace is the sum of the diagonal elements. Equation (8) was used to calculate lack of fit (lof). In this case, **F**_**exp**_ = **A**_**exp**_; **F**_**calc**_ = **A**_**calc**_. Second, (8) was used to compare the distribution diagrams calculated by application of conventional stepwise chemical model from the ones simulated by the cooperative model. In this case, a concentration matrix **F**_**exp**_ = **C**_**DNAL**_ was calculated using a model, and a matrix **F**_**calc**_ = **C**_**calc**_ was calculated in turn by using (3) on the last LS iterative step. In all cases, Matlab-2014® software was used in the calculations.

## 3. Results and Discussion

### 3.1. Cooperative Binding (Data Matrix** A**_**1a**_)

The UV-Vis absorption spectra changes of an aqueous solution in binding reactions of TMPyP4 and G4 in all known cases are typically similar. To simulate data set one have to construct bathochromic red shifts 15–20 nm and hypochromicities at *λ*_max_ (60–70%) of the Soret band were observed during coordination TMPyP4 to G4. Spectra **S**_**L**_ and **S**_**Mon**_ used to build the data sets **A**_**exp**_ are shown in Supplementary Material (Figure S1). In the simplest case, we assume that spectrum **S**_**MonL**_ is distorted superposition of **S**_**L**_ and **S**_**Mon**_. This situation is a binding without spectral interference. More general case of spectral interference (SI) is the mutual perturbation of the ligand spectra bound in the vicinity.

Let us consider the model defined by equilibrium constants $\mathrm{lg}K_{1\text{-}2}=\left[\begin{array}{cc} 6.5 & 6.5 \end{array}\right]$ and **ω** = 2.0, governing the formation of a Mon*L* species (two equal sites plus a positive binding cooperativity between sites). Simulated data matrix (see Supplementary Material, Figure S2) **A**_**exp**_ should be written as

(9) $$\begin{array}{c} A_{exp}=\left[\;L\;\right]S_{L}+C_{MonL}S_{M}+\tau . \end{array}$$

Here elements of the matrix **τ** are put to zero; **S**_**L**_ is molar absorptivities of free ligand. Given that two sites are equivalent and have no SI the matrix of molar absorptivities of free and occupied sites I-II is $S_{M}={\left[\begin{array}{cccc} S_{Mon_{1}} & S_{Mon_{2}} & S_{Mon_{1}L} & S_{Mon_{2}L} \end{array}\right]}^{T}$. The concentration matrix used to calculate the absorbance data matrix is

(10) $$\begin{array}{c} C_{MonL}=\left[\begin{array}{cccc} \left[\text{Mo}{\text{n}}_{1}\right] & \left[\text{Mo}{\text{n}}_{2}\right] & \left[\text{Mo}{\text{n}}_{1}L\right]+\left[\text{Mo}{\text{n}}_{1}L2\right] & \left[\text{Mo}{\text{n}}_{2}L\right]+\left[\text{Mo}{\text{n}}_{2}L2\right] \end{array}\right]. \end{array}$$

SVD applied for **A**_1**a**_ yields *R* = 3 indicating the three optically active chemical species in the full spectrum and local rang *R*_*L*_ = 2 indicating the species in spectral window of TMPyP4.

To fit the data, in accordance with proposed approach, the conventional noncooperative model of stepwise complex formation first has been tested.

(11) G4+TMPyP4⟷G4TMPyP4G4TMPyP4+TMPyP4⟷G4TMPyP42

Here stepwise equilibrium constants *x*(1) = lg⁡**K**_1_ and *x*(2) = lg⁡**K**_2_ are independent variables and *ω* = constant = 1. However the LS fitting gives a poor fit to the target matrix **A**_**exp**_. The reproduced **A**_1**a**_ matrix is computed as

(12) $$\begin{array}{c} A_{calc}=\left[\;L\;\right]S_{L}+C_{DNAL}S_{F}+\tau . \end{array}$$

Here $S_{F}={\left[\begin{array}{ccc} S_{DNA} & S_{DNAL} & S_{DNAL_{2}} \end{array}\right]}^{T}$; *τ* is lof. Once we analysed simulated data matrix **A**_1**a**_ using a model of independent binding sites, taking into account the existence of 2 sites of 2 types, the best fit was model with two equivalent binding sites $\mathrm{lg}K_{1\text{-}2}=\left[\begin{array}{cc} 7.04 & 7.04 \end{array}\right]$, and a lof is 0.2%.

**Figure 1:**
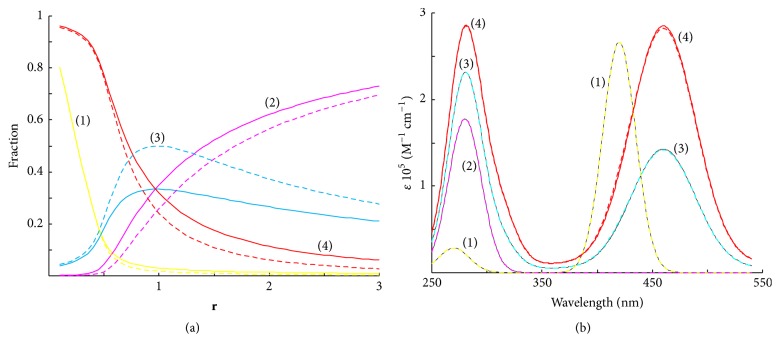
Distribution diagram (a) and pure species spectra (b) resolved by deconvolution of **A**_1**a**_. Species fractions calculated as a function of the DNA/ligand molar ratio *r* (*r* = *C*_*D*_/*C*_*L*_; *C*_*L*_ = 5 *μ*mol/L) in accordance with the model: $\mathrm{lg}K_{1\text{-}2}=\left[\begin{array}{cc} 7.04 & 7.04 \end{array}\right]$, *ω* = 1 = const, shown as dotted lines. Profiles simulated in accordance with a binding model: $\mathrm{lg}K_{1\text{-}2}=\left[\begin{array}{cc} 6.5 & 6.5 \end{array}\right]$, *ω* = 2.0, shown as solid lines. (1)  *L*, (2) DNA, (3) DNA*L*, and (4) DNA*L*_2_.

Figure 1(a) shows distribution diagrams of hypothetical DNA oligomer between species DNA, DNAL, and DNAL_2_. As can be seen from Figure 1(a), the resolved distribution of the species significantly differs from the theoretical one (**PE** 18.4%).  Figure 1(b) shows simulated and resolved pure species spectra. The shape of spectral profiles is due to calculated monomers spectra. The spectrum of species DNA*L*_2_ is doubled spectrum of DNA*L*, 2**S**_**DNAL**_ = **S**_**DNAL**_2__.

*RAFA of the Data Matrix A_1a_*. Given that spectra **S**_**DNA**_ and **S**_**L**_ are easily measured experimentally, we can calculate the matrix **A**′.

(13) $$\begin{array}{c} A^{\prime }=A_{exp}-\left[\;L\;\right]S_{L}-\left[\;DNA\;\right]S_{DNA}+\tau . \end{array}$$

With the result of (13), **A**_**exp**_ can be divided into two parts, where the known species *L* and DNA only contribute to the one part. The other part **A**′ contains linear combination of the unknown spectra. The SVD of matrix **A**_1**a**_′ indicated no rank annihilation of **A**_1**a**_ including concentration window of the ligand. This result conforms that spectral species are inaccurately recognized.

The difference between theoretical and calculated fractions of free sites and these bound with a ligand, that is, Mon_1_, Mon_2_, Mon_1_*L*, Mon_2_*L*, Mon*L*_2_, also can be seen. However, the concentration of free monomer is calculated correctly. The matrix **A**′′ may be written in the form:

(14) A′′=Aexp−LSL−Mon1Mon2SMon1SMon2T+τ.

The reduced data matrix **A**_1**a**_′′ has been obtained by subtracting the contribution of the proposed model species from the complete data set. The rank of the matrix **A**_1**a**_′′ is less than the rank of the full **A**_1**a**_ matrix *R*′′ = *R* − 1. So in the full spectral range one species is correctly recognized.

The rank annihilation completes the conclusion that sites are indistinguishable monomers.

### 3.2. Anticooperative Binding without SI (Data Matrix** A**_**1b**_)

To simulate a data matrix **A**_1**b**_ obeying (9), the same spectra as in the previous example were used (see Figure S1). Concentrations are calculated from the model $\mathrm{lg}K_{1\text{-}2}=\left[\begin{array}{cc} 6.5 & 6.5 \end{array}\right]$, **ω** = 0.3. Fitting the absorbance data matrix using conventional stepwise model without cooperativity gives the **lof**(**A**_1**b**_) ≈ 0.0%. Here the best fit gives model considering 2 sites of 2 different types $\mathrm{lg}K_{1\text{-}2}=\left[\begin{array}{cc} 6.79 & 5.16 \end{array}\right]$. The stepwise model provides a good fit to simulated data. Consequently, we have a model ambiguity as a result of the rotational ambiguity. All species fractions quantified very well (see Table 1).

*RAFA of the Data Matrix A_1b_*. As can be seen from Table 1, SVD is applied on **A**_1**b**_, **A**_1**b**_′, and **A**_1**b**_′′ giving the different result as for the above case of positive cooperativity. One can see the rank annihilation in full spectral range and in a spectral window of ligand. This result suggests that subtracted spectral species are correctly recognized. The rank of the matrix **A**_1**b**_′′ is less than the rank of the full **A**_1**b**_′ matrix *R*′′ = *R*′ − 1. So in the full spectral range extra one species is correctly recognized.

### 3.3. Cooperative Binding with SI (Data Matrix** A**_**1c**_)

In the previous examples, we have used an assumption that all G4-ligand complexes formed possess the same absorbance spectrum, irrespective of the binding mode. However this assumption is violated in most actual systems, probably in each system with multiple binding sites. We will use SI when binding in each site leading to a change in the spectrum of ligand in the vicinity. Given that bound ligands have SI, an experimental matrix **A**_1**c**_ should obey (12) where the matrix of molar absorptivities is $S_{M}={\left[\begin{array}{cccccc} S_{Mon_{1}} & S_{Mon_{2}} & S_{Mon_{1}L} & S_{Mon_{2}L} & S_{Mon_{1}L2} & S_{Mon_{2}L2} \end{array}\right]}^{T}$ (see Supplementary Materials, Figure  S1b) and

(15) $$\begin{array}{c} C_{MonL}=\begin{array}{c} \begin{array}{c} \begin{array}{cccccc} \left[\left[\text{Mo}{\text{n}}_{1}\right]\right. & \left[\text{Mo}{\text{n}}_{2}\right] & \left[\text{Mo}{\text{n}}_{1}L\right] & \left[\text{Mo}{\text{n}}_{2}L\right] & \left[\text{Mo}{\text{n}}_{1}L2\right] & \left.\left[\text{Mo}{\text{n}}_{2}L2\right]\right] \end{array} \end{array} \end{array} \end{array}$$

is concentration matrix calculated from the before used binding model $\mathrm{lg}K_{1\text{-}2}=\left[\begin{array}{cc} 6.5 & 6.5 \end{array}\right]$, **ω** = 2.0. The distribution plot of resolved oligomer species is very similar to these shown on Figure 1. SVD is applied on **A**_1**c**_ yielding four main factors defining absorbance variance (*R* = 4), and the intrinsic absorption of the ligand is determined by three factors (*R*_*L*_ = 3). With the application of (13), the rank annihilation was not achieved. This suggests that all forms are calculated with poor accuracy. Application of (14) for total spectral range gives *R*′′ = 3 which means the correct calculation of only the monomer contribution.

### 3.4. Anticooperative Binding with SI (Data Matrix** A**_**1d**_)

**Figure 2:**
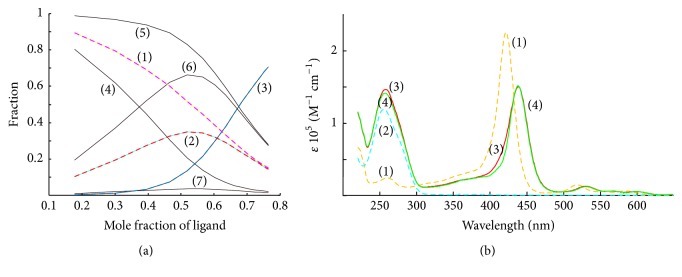
Distribution diagram (a) and pure species spectra (b) resolved by deconvolution of **A**_3**e**_. Dashed lines are calculated in accordance with anticooperative model: $\mathrm{lg}K_{1\text{-}2}=\left[\begin{array}{cc} 6.79 & 6.79 \end{array}\right]$, *ω* = 0.43; solid lines are calculated in accordance with stepwise model $\mathrm{lg}K_{1\text{-}2}=\left[\begin{array}{cc} 7.07 & 5.79 \end{array}\right]$, *ω* = 1.0; (a) (1)  [Mon_1_]/*C*_*D*_ = [Mon_2_]/*C*_*D*_, (2)  [Mon_1_*L*]/*C*_*D*_ = [Mon_2_*L*]/*C*_*D*_, (3)  [Mon_1_*L*2]/*C*_*D*_ = [Mon_2_*L*2]/*C*_*D*_, (4)  [Mon_1_]/*C*_*D*_, (5)  [Mon_2_]/*C*_*D*_, (6)  [Mon_1_*L*]/*C*_*D*_, (7)  [Mon_2_*L*]/*C*_*D*_; (b) (1) TMPyP4, **S**_**L**_; (2)  Mon_1_ = Mon_2_, **S**_**Mon**_; (3)  Mon_1_*L*, **S**_**Mon**_1_**L**_; (4)  Mon_2_*L*, **S**_**Mon**_2_**L**_; pH 7.1, *T* = 25°C.

Consider the binding model $\mathrm{lg}K_{1\text{-}2}=\left[\begin{array}{cc} 6.5 & 6.5 \end{array}\right]$, **ω** = 0.3. The model is used to simulate anticooperative binding with SI. In this example, the model ambiguity is the result of LS fitting. The presence of SI does not affect the shape of oligomer distribution diagram shown on Figure 2. However, the result of RAFA is not the same. The identification of sites identity is ambiguous in this case. As can be seen from Table 1, the result of SVD application on matrices **A**_1**d**_, **A**_1**d**_′, and **A**_1**d**_′′ is the same as for these for two types of sites with different spectral characteristics (see below).

### 3.5. The Binding to G4 DNA with Different Sites (Data Matrix** A**_**2a**_)

Consider lastly an oligomer which has different binding sites. Consequently each site should have different affinities for the ligand and may have a different absorbance signal. The model with a strong binding site and weaker binding site $\mathrm{lg}K_{1\text{-}2}=\left[\begin{array}{cc} 7 & 6 \end{array}\right]$ to simulate the data matrix was used. Simulated data can be decomposed using the model of two equivalent binding sites and anticooperativity $\mathrm{lg}K_{1\text{-}2}=\left[\begin{array}{cc} 6.74 & 6.74 \end{array}\right]$, **ω** = 0.58 with excellent precision (see Table 1). In this case to simulate data use (12), but reproduced data matrix is calculated in accordance with (9). Resolved distribution of G4 oligomer species well reproduce those used for simulation. The rank of a data matrix constructed in accordance with (12) is equal to the rank of **C** (*R* = 4). The rank of both **A**_2**a**_ and **A**_2**b**_ in spectral window (*R*_*L*_ = 3) is less than the number of spectral species in full spectral range because the light absorption by monomer species is zero in this interval. Nevertheless, the rank of **A**′ along with **A**′′ two units less compared with **A** can be seen due to rotational ambiguity. Moreover we can see the same sequences of *R* (*R*_*L*_), *R*′ (*R*_*L*_′), and *R*′′ (*R*_*L*_′′) regardless whether or not there is spectral MI. Thus, given example of binding shows inapplicability of* RAFA* to indicate the fact that sites are different or the same sites bind in anticooperative fashion.

### 3.6. The TMPyP4 Binding with Nucleotide Sequence 5′-(CG3)2CGC(AG3)2G-3′ Experimental Data (Matrix** A**_**3e**_)

To illustrate the applicability of the suggested approach to model porphyrin binding with a macromolecule, a porphyrin TMPyP4 binding to 5′-(CG3)2CGC(AG3)2G-3′ was selected as experimental example. UV-absorbance experimental data published previously [15] has been used for further numerical treatment. In the original work [15] mole-ratio experiments to study the G4-ligand interaction were performed. When solution of G4 is added to solution of TMPyP4 in buffer pH 7.1, at 25°C the induced bathochromic red shifts and hypochromicities for the Soret band clearly indicate the DNA-ligand complexation. A stepwise complex formation model has been applied in [15] to interpret the experimental data (matrix **A**_3**e**_). Conventional stepwise complex formation algorithm best fits the data matrix which included one strong binding site and one weak binding site ($\mathrm{lg}K_{1\text{-}2}=\left[\begin{array}{cc} 7.07 & 5.79 \end{array}\right]$), **lof**(**A**_3**e**_) ≈ 0.25%. However, the same fit gives a model with two equivalent anticooperative binding sites. The calculated intrinsic binding constants **K**_1-2_ and the cooperativity parameter **ω** are summarized in Table 2. The calculated distribution diagrams for this mole-ratio spectrometric titration of G4 by TMPyP4 for both models are compared in Figure 2. For this G4 DNA example of binding interaction, the LS fitting discloses a model ambiguity. The values of *R*′′ (*R*_*L*_′′) we have given in Table 2 equal the values of all these quantities which are given in Table 1 for anticooperative binding with identical sites without SI. Based on this observation, one can suggest a model of anticooperative assembling of TMPyP4 and G4 DNA with identical binding sites without SI. In addition, the calculated pure spectra for species involved in the equilibrium are shown in Figure 2. The spectra are calculated according to the following equation:

(16) S=A3e′′Mon1L+Mon2LMon1L2+=A3e′Mon1L+Mon1L2Mon2L+Mon2L2+.

The spectra associated with the ligand are compared in Figure 2(b). Some difference between Mon_1_*L* and Mon_2_*L* spectra can be seen. But this very little difference is probably due to insufficiency of free sites and free ligand fixed spectra measurements. The calculation of pure absorption monomers spectra is impossible because of rank deficiency after subtracting contribution of free ligand absorbance from full data matrix **A**_3**e**_ (see Table S2 in the Supplementary Material).

In summary, the methodology of the absorbance data matrix numerical treatment for G4 DNA/ligand equilibrium system has been successfully developed using a hard modelling matrix method and Rank Annihilation Factor Analysis. Accurate identification of binding sites identity and the mutual influence between bound ligands, which is essential to G4 DNA* in vivo* function annotation, can be done for sites involved in cooperative ligand binding. Unfortunately the discovery of the sites identity is hindered when sites are involved in anticooperative binding. It is shown that the spectrometrically measured titration curves can be described ambiguously using the model of two different and independent binding sites as well as that of two mutually influencing identical sites in the case of anticooperative binding.

## Supplementary Material

Figure S1. The graph of Gaussians used to build the data sets simulating the G4-ligand interaction.
Figure S2. Simulated data matrix **A1a** (UV-Vis absorbance spectra) in accordance with the model: lg**K1**_-_**2 **= [6.5 6.5] and **ω **= 2.0.
Table S1. Spectroscopically and ITC determined data of TMPyP4 binding to G4 DNA.
Table S2. Results of analysis of UV-Vis absorption data matrix **A3e** by the PCA method, and results of subtraction of the contribution of the spectral species.

## Figures and Tables

**Table 1:** Intrinsic binding constants calculated for simulated ligand G4 DNA equilibrium systems.

	Data matrix	lg⁡**K**_1-2_, **ω** theoretical model	lg⁡**K**_1-2_, *ω* LS calc. parameters	**lof** %	**P** **E**_**F**_ %	**P** **E**_**S**_ %	*R* (*R*_*L*_)	*R*′ (*R*_*L*_′)	*R*′′ (*R*_*L*_′′)
*Identical sites*									
Cooperative binding without SI	**A**_1**a**_	$\left[\begin{array}{cc} 6.5 & 6.5 \end{array}\right]$, 2.0	$\left[\begin{array}{cc} 7.04 & 7.04 \end{array}\right]$, 1.0^*∗*^	0.2	18.4	1.0	3 (2)	3 (2)	2 (2)
Anticooperative binding without SI	**A**_1**b**_	$\left[\begin{array}{cc} 6.5 & 6.5 \end{array}\right]$, 0.3	$\left[\begin{array}{cc} 6.79 & 5.16 \end{array}\right]$, 1.0^*∗*^	≈0^*∗∗*^	≈0^*∗∗*^	≈0^*∗∗*^	3 (2)	2 (1)	1 (1)
Cooperative binding with SI	**A**_1**c**_	$\left[\begin{array}{cc} 6.5 & 6.5 \end{array}\right]$, 2.0	$\left[\begin{array}{cc} 6.75 & 6.75 \end{array}\right]$, 1.0^*∗*^	0.8	19.4	2.3	4 (3)	4 (3)	3 (3)
Anticooperative binding with SI	**A**_1**d**_	$\left[\begin{array}{cc} 6.5 & 6.5 \end{array}\right]$, 0.3	$\left[\begin{array}{cc} 6.79 & 5.16 \end{array}\right]$, 1.0^*∗*^	≈0^*∗∗*^	≈0^*∗∗*^	≈0^*∗∗*^	4 (3)	2 (2)	2 (2)

*Different sites*									
Binding without SI	**A**_2**a**_	$\left[\begin{array}{cc} 7 & 6 \end{array}\right]$, 1	$\left[\begin{array}{cc} 6.74 & 6.74 \end{array}\right]$, 0.58	≈0^*∗∗*^	≈0^*∗∗*^	≈0^*∗∗*^	4 (3)	2 (2)	2 (2)

*R* is the rank of full data matrix **A**; *R*_*L*_ is the rank of data matrix part covering spectral window of a ligand; SI is spectral interference; ^*∗*^parameter is fixed; ^*∗∗*^lof < 10^−4^≈ 0; *C*_*L*_ + *C*_*P*_ = 5 *μ*M.

**Table 2:** Intrinsic binding constants for TMPyP4 binding to G4 oligomer calculated from experimental UV-Vis titrations (pH 7.1, *T* = 25°C).

A model	Data matrix	lg⁡**K**_1-2_, **ω** LS calculated parameters	**lof** **%**	**P** **E**_**F**_ ***%***	rank **A** *R* (*R*_*L*_)	rank **A**′ *R*′ (*R*_*L*_′)	rank **A**′′ *R*′′ (*R*_*L*_′′)
Stepwise complex formation	**A**_3**e**_	$\left[\begin{array}{cc} 7.07 & 5.79 \end{array}\right]$, 1.0^*∗*^	0.25	≈0^*∗∗*^	3 (2)	2 (1)	—
Cooperative binding	**A**_3**e**_	$\left[\begin{array}{cc} 6.79 & 6.79 \end{array}\right]$, 0.43	0.25	≈0^*∗∗*^	—	—	1 (1)

*R* is the rank of full data matrix **A**; *R*_*L*_ is the rank of data matrix part covering spectral window of a ligand 340–540 nm.
