# Supplementary material for: The Evidence of Cooperative Binding of a Ligand to G4 DNA
Source: J Anal Methods Chem. 2017 Oct 18;2017:6780521. doi: 10.1155/2017/6780521 (PMC5664379; doi:10.1155/2017/6780521)
Supplement: Supplementary file 1 — Figure S1. The graph of Gaussians used to build the data sets simulating the G4-ligand interaction. Figure S2. Simulated data matrix A1a (UV-Vis absorbance spectra) in accordance with the model: lgK1-2 = [6.5 6.5] and ω = 2.0. Table S1. Spectroscopically and ITC determined data of TMPyP4 binding to G4 DNA. Table S2. Results of analysis of UV-Vis absorption data matrix A3e by the PCA method, and results of subtraction of the contribution of the spectral species. [file 6780521.f1.doc]

**Supplementary Information**

**The evidence of cooperative binding a ligand to G4 DNA**

© 2017 A.G. Kudrev

Saint Petersburg State University, St.Petersburg State University, 7/9 Universitetskaya nab., St. Petersburg, 199034 Russia.

E-mail: [kudrevandrei@mail.ru](mailto:kudrevandrei@mail.ru)

250

300

350

400

450

500

0

0.5

1

1.5

2

2.5

3

ε∙10^5^ (cm^-1^M^-1^)

a

1

3

2

1

3

250

300

350

400

450

500

0

0.5

1

1.5

2

2.5

3

b

1

3

2

ε∙10^5^ (cm^-1^M^-1^)

SI

1

3

Wavelength, nm.

Wavelength, nm.

Figure S1. Pure absorbance spectra used to build the data sets simulating the ligand - G4 interaction (2 equal sites). a. without spectral interference; 1- free ligand, ***S_L_***; 2- free site, ***S_Mon_***; 3- site with bound ligand, ***S_MonL_***. b. the perturbation of bound ligand spectrum; 1- free ligand, ***S_L_***; 2- free site, ***S_Mon_***; 3- site with bound ligand, ***S_MonL_***. SI (change of a spectrum when bound second ligand) are given as dotted line.

A spectrum of a pure species was calculated by formula:

$$S=\frac{e^{-(X-\mu)^{2}/2\sigma^{2}}}{\sigma\sqrt{2\pi}}+\frac{e^{-(X'-\mu')^{2}/2\sigma'^{2}}}{\sigma'\sqrt{2\pi}}$$

Here ***S*** is the matrix of molar absorptivities (molar extinction coefficients, cm^-1^M^-1^), parameters (*X, µ, σ* and *X’, µ’, σ’*) were chosen to simulate spectral changes (induced bathochromic red shifts (15-20 nm), and hypochromicities (60-70%) for the Soret band) reported previously for porphyrin tetraplex binding. The percent of hypochromicity at a wavelength (h%) of porphyrin Soret band was calculated using formula:

h%=[(ε_f_ - ε_b_)/ε_f_]∙100

where ε_f_ and ε_b_ are molar absorptivities of free and bound ligand correspondingly.

**Abs _500 nm_**

**Abs**

250

300

350

400

450

500

550

0

0.5

1

1.5

2

2.5

3

0

0.5

1

1.5

2

2.5

3

0

0.05

0.1

0.15

0.2

0.25

b

a

Wavelength, nm.

*r*

Figure S2. a. Simulated UV-Vis absorbance spectra (data matrix ***A_1a_*** ) in accordance with the model: lg**K_1_**-**_2_** = [6.5 6.5] and **𝜔** = 2.0.

b. The dependence of absorbance at 500 nm vs. molar ratio 𝑟 (𝑟 = 𝐶𝐷/𝐶𝐿).

Table S1. Spectroscopically and ITC determined data of TMPyP4 binding to G4 DNA.

| G4 DNA | n | lgK_b_ | Method | Remarks | Ref. |
| --- | --- | --- | --- | --- | --- |
| [d(G_2_T_2_G_2_TGTG_2_T_2_G_2_)] | n_1_ = 1 | 5.25 | ITC | pH 7, 25^o^C,  K^+^-BPES | [1S] |
| [d(G_2_T_2_G_2_TGTG_2_T_2_G_2_)] | n_1_ = 1.2 | 5.34 | UV-VIS | pH 7, 25^o^C,  K^+^-BPES | [1S] |
| [d(T_4_G_4_)]_4_ | n_1_ = 2.9 | 4.89 | ITC | pH 7, 25^o^C,  K^+^-BPES | [1S] |
| [d(T_4_G_4_)]_4_ | n_1_ = 2.6 | 5.23 | UV-VIS | pH 7, 25^o^C,  K^+^-BPES | [1S] |
| [d(T_4_G_4_)]_4_ | n_1_ = 0.9 | 6.63 | UV-VIS | pH 7, 25^o^C,  Na^+^-BPES | [1S] |
| [d(AG_3_(T_2_AG_3_)_3_)] | n_1_ = 1.9 | 4.45 | ITC | pH 7, 25^o^C,  K^+^-BPES | [1S] |
| [d(AG_3_(T_2_AG_3_)_3_)] | n_1_ = 1.8 | 4.87 | UV-VIS | pH 7, 25^o^C,  K^+^-BPES | [1S] |
| [d(AG_3_(T_2_AG_3_)_3_)] | n_1_ = 1.55  n_1_ = 1.2 | 6.03  8.65 | UV-VIS | pH 7.5,  150 mM K^+^ | [2S] |
| [d(AG_3_(T_2_AG_3_)_3_)] | n_1_ = 1.48  n_1_ = 0.54 | 6.94  8.35 | UV-VIS | pH 7.5,  150 mM K^+^,  40% PEG | [2S] |
| [d(AG_3_(T_2_AG_3_)_3_)] | n_1_ = 4 | -- | UV-VIS | pH 7.5, Tris-HCl;  100 mM Na^+^ | [3S] |
| [d(TAG_3_(T_2_AG_3_)_3_T)] | n_1_ = 6.1  (n_1_ ≈ 5) | 6.81 | UV-VIS;  (CD; FL) | pH 7.2, 25^o^C,  Tris-HCl | [4S] |
| [d(TAG_3_(T_2_AG_3_)_3_T)] | n_1_ = 4.6  (n_1_ ≈ 4.9) | 6.80 | UV-VIS;  (CD; FL) | pH 7.2, 25^o^C,  Tris-HCl,  10 mM K^+^ | [4S] |
| [d(TAG_3_(T_2_AG_3_)_3_T)] | n_1_ = 2.7  (n_1_ ≈ 2.8) | 6.25 | UV-VIS;  (CD; FL) | pH 7.2, 25^o^C,  Tris-HCl,  100 mM K^+^ | [4S] |
| [d(G_3_(T_2_AG_3_)_3_)] | n_1_ = 1.1  n_2_ = 1.7 | 6.70  6.04 | UV-VIS | pH 7.0, 25^o^C,  100 mM K^+^ | [5S] |
| [d(G_3_(T_2_AG_3_)_3_)] | n_1_ = 1  n_2_ = 2 | 6.60  5.70 | ITC | pH 7.0, 25^o^C,  100 mM K^+^ | [5S] |
| [d(G_3_T) _3_G_3_)] | n_1_ = 0.8  n_2_ = 1.0 | 7.85  6.32 | UV-VIS | pH 7.0, 25^o^C,  100 mM K^+^ | [6S] |
| [d(G_3_T)_3_T_2_G_3_)] | n_1_ = 0.7  n_2_ = 2.0 | 8.20  6.30 | UV-VIS | pH 7.0, 25^o^C,  100 mM K^+^ | [6S] |
| [d(G_3_T)_3_T_4_G_3_)] | n_1_ = 1.2  n_2_ = 2.2 | 8.75  6.40 | UV-VIS | pH 7.0, 25^o^C,  100 mM K^+^ | [6S] |
| [d(AG_3_(T_2_AG_3_)_3_)] | n_1_ = 2 | 7.6 ± 0.9 | UV-VIS | pH 7.2,  Direct Fit | [7S] |
| [d(AG_3_(T_2_AG_3_)_3_)] | n_1_ = 2.8 | 2.5 ± 0.6 | UV-VIS | pH 7.2, Scatchard  binding model | [7S] |

n_1_ and n_2_ are the phenomenological stoichiometries; lgK_b_, M^-1^ is equilibrium binding constant; UV-VIS – is UV-visible optical spectroscopy; ITC is isothermal titration calorimetry; SP is spectrophotometry; K^+^ (Na^+^)-BPES is K(Na)H_2_PO_4_/K(Na)_2_HPO_4_, K(Na)Cl, EDTA buffer; PEG is poly(ethylene glycol); FL is fluorescence;

[1S] I. Haq, J.O. Trent, B.Z. Chowdhry, T.C. Jenkins, J. Am. Chem. Soc. 121 (1999) 1768.

[2S] C. Wei, G. Jia, J. Zhou, G. Han, C. Li, Phys. Chem. Chem. Phys. 11 (2009) 4025.

[3S] C. Wei, G. Jia, J. Yuan, Z. Feng, C. Li, Biochemistry 45 (2006) 6681.

[4S] H.J. Zhang, X.F. Wang, P. Wang, X.C. Ai, J.P. Zhang, Photochem. Photobiol. Sci. 7

(2008) 948.

[5S] A. Arora, S. Maiti, J. Phys. Chem. B 112 (2008) 8151.

[6S] A. Arora, S. Maiti, J. Phys. Chem. B 113 (2009) 8784.

[7S] T.L. Ruan, S.J. Davis, B.M. Powell, C.P. Harbeck, J. Habdas, P. Habdas,

L.A. Yatsunyk, Lowering the overall charge on TMPyP4 improves its selectivity for G-quadruplex DNA, Biochimie (2016), doi: 10.1016/j.biochi.2016.11.003.

Table S2. Results of analysis of UV absorption data matrix ***A_3e_*** by the PCA method, and results of subtraction of the contribution of the spectral species (RAFA).

| Data mat-rix | Eigenvalue of  Cov( ***A***)  (Variance captured this PC %) | Eigenvalue of  Cov( ***A_L_***)  (Variance captured this PC %) | Eigenvalue of  Cov( ***A'***)  (Variance captured this PC %) | Eigenvalue of  Cov( ***A'_L_***)  (Variance captured this PC %) | Eigenvalue of  Cov( ***A''***)  (Variance captured this PC %) | Eigenvalue of  Cov( ***A''_L_***)  (Variance captured this PC %) |
| --- | --- | --- | --- | --- | --- | --- |
| ***A_3e_*** | **38.9 (95.16)**  **1.93 (4.71)**  **5.32e-2(0.13)**  3.26e-5(0.00)  3.76e-6(0.00) | **16.2(99.13)**  **1.42e-1(0.87)**  1.62e-4(0.00)  1.08e-5(0.00)  2.61e-6(0.00) | **23.3 (99.72)**  **6.50e-2(0.28)**  9.52e-5(0.00)  7.71e-5(0.00)  1.62e-5(0.00) | **6.96 (99.98)**  **1.70e-3(0.02)**  4.49e-5(0.00)  1.65e-5(0.00)  4.02e-6(0.00) | **16.6 (99.99)**  **1.78e-3(0.01)**  1.06e-4(0.00)  4.19e-5(0.00)  1.16e-5(0.00) | **6.91 (99.98)**  **1.51e-3(0.02)**  4.18e-5(0.00)  1.98e-5(0.00)  3.03e-6(0.00) |
|  |  |  |  |  |  |  |
